# Supplementary material for: A new target: AlkBH2 promotes bladder cancer by upregulation of inflammation
Source: PLoS One. 2026 May 7;21(5):e0348059. doi: 10.1371/journal.pone.0348059 (PMC13152141; doi:10.1371/journal.pone.0348059)
Supplement: S1 Table — (A) The manufacturer’s name and product number of the key reagents of the ELISA kit. (B) The primer sequence of AlkBH2 mRNA and the silencing sequence of AlkBH2. (C) The dilution concentration of the antibody reagent, as well as the manufacturer’s name and product number. (D) The manufacturer’s name and product number of relevant agents. (DOCX) [file pone.0348059.s001.docx]

**S1. Table**

**A**

**ELISA used for cells**

| **Name** | **Company** | **Catalog Number** |
| --- | --- | --- |
| IL-1β | Proteintech, Wuhan, China | KE00021 |
| TNF-α | Thermo Fisher Scientific, Massachusetts, USA | ECH009 |
| IL-12 | Thermo Fisher Scientific, Massachusetts, USA | KAC1568 |
| IL-17 | Thermo Fisher Scientific, Massachusetts, USA | EH260RB |
| IL-10 | Thermo Fisher Scientific, Massachusetts, USA | 88-7106-88 |
| IL-4 | Proteintech, Wuhan, Chian | KE00016 |
| TGF-β | Proteintech, Wuhan, China | KE00002 |
| IL-37 | Thermo Fisher Scientific, Massachusetts, USA | 88-52103-22 |

**B**

**Sequences of AlkBH2**

| **Gene** | **Sequences** |
| --- | --- |
| AlkBH2 forward primer | 5’-TCCTCTTCCGGCACAAAGAC-3’ |
| AlkBH2 reverse primer | 5’-GGGGTGGTTCATCATCAGCA-3’ |
| AlkBH2 silencing | 5’-TGGACAGATTCCTGGTGAAAG-3’ |

**C**

**Antibodies used for Western blotting**

| **Antibody** | **Company** | **Catalog Number** | **RRID** | **Solution** |
| --- | --- | --- | --- | --- |
| AlkBH2 | Proteintech, Wuhan, China | 17809-1-AP | AB_3085541 | 1:1000 |
| NRF2 | Boster, Wuhan, Chian | A00078-1 | AB_3080932 | 1:500 |
| HO-1 | Proteintech, Wuhan, China | 10701-1-AP | AB_2118685 | 1:1000 |
| P-P65 | Abcam, UK | AB109458 | AB_10864939 | 1:1000 |
| Actin | Servicebio, Wuhan, Chian | GB11001-100 | AB_2305186 | 1:500 |
| P56 | Abcam, UK | AB16502 | AB_443394 | 1:1000 |
| Lamin B1 | Boster, Wuhan, Chian | PB9611 | AB_3082187 | 1:500 |

**D**

**Relevant reagents**

| **Name** | **Company** | **Catalog Number** |
| --- | --- | --- |
| DMEM | Thermo Fisher Scientific, Massachusetts, USA | 12491015 |
| FBS | Thermo Fisher Scientific, Massachusetts, USA | 10099141C |
| Penicillin-streptomycin | Thermo Fisher Scientific, Massachusetts, USA | 15070063 |
| Hematoxylin | Servicebio, Wuhan, Chian | G1004 |
| Eosin | Servicebio, Wuhan, Chian | G1001 |
| Triton X-100 | Servicebio, Wuhan, Chian | GC204003 |
| Goat serum | Servicebio, Wuhan, Chian | G1208 |
| Alexa Fluor® 488 | Servicebio, Wuhan, Chian | GB25303 |
| RNA Purification Kit | Thermo Fisher Scientific, Massachusetts, USA | K0732 |
| cDNA Synthesis Kit | Tsingke Biotechnology, Beijing, China | TSK302S |
| Master qPCR Mix | Tsingke Biotechnology, Beijing, China | TSE201 |
| HRP | Proteintech, Wuhan, China | RGAR001 |
| Chemiluminescent reagents | Thermo Fisher Scientific, Massachusetts, USA | 34580 |
| Cell cycle kit | Beyotime, Shanghai, China | C1052 |
| Mitomycin C | MCE, New Jersey, USA | HY-13316 |
| Matrigel | Corning, USA | 356234 |
| EGM-2 bulletkit | Lonza, Basel, Switzerland | CC-3156 |
