# Supplementary material for: A new target: AlkBH2 promotes bladder cancer by upregulation of inflammation
Source: PLoS One. 2026 May 7;21(5):e0348059. doi: 10.1371/journal.pone.0348059 (PMC13152141; doi:10.1371/journal.pone.0348059)
Supplement: Attachment — Submitted filename: Response to Reviewers.docx [file pone.0348059.s004.docx]

Dear editor,

Thank you for your suggestions. We will carefully revise each suggestion and provide responses.

1. We have revised the manuscript in accordance with the formatting requirements of the PLOS ONE journal.

2. “The funders had no role in study design, data collection and analysis, decision to publish, or preparation of the manuscript.” We have added this Role of Funder statement in cover letter.

3. We have removed any funding-related text from the manuscript.

4. We have removed any ethics statement text besides the Methods from the manuscript.

5. According to Supporting Information guidelines, we have amended the caption.

6. We have uploaded original uncropped and unadjusted images underlying all blot or gel results reported in Supporting Information, and note this information in cover letter.

To reviewer1

Thank you for your all suggestions, which is extremely valuable and has greatly benefited us.

1. Thank you very much for your guidance. Due to the limitations of technology, your suggestions made us realize for the first time that there are so many methods for detecting cell proliferation. We have supplemented the real-time cell analysis experiment to replace CCK-8.

2. Thank you very much for your reminder. We did add mitomycin in the scratch test, but due to our writing oversight, it was not described in the methods section. We have already made the necessary revisions.

3. Thank you very much for your suggestion. As the results of proteomics sequencing were not satisfactory, we did not include them. Now, following your advice, we have added the volcano plot, heat map, and enrichment plot, among other results, to the figures.

4. Thank you very much for your suggestion. In order to ensure that the NF-κB nuclear localization mechanism is more strictly regulated, we have taken your advice into account and conducted additional experiments. We extracted nuclear proteins and used Lamin B1 as the marker to assess the relative nuclear entry of NF-κB.

5. Thank you for your comment regarding the transfection rate. This was our negligence in method as described. We have now added the method for calculating the transfection rate. First, count the cells using white light, then in the same field of view, calculate the GFP-positive cells using fluorescence, and the resulting ratio is the transfection rate.

6. Thank you for your comments regarding the inhibitor of AlkBH2. We fully agree with your suggestions. However, we are unable to supplement this experiment due to the time and economic costs involved. Although this may affect the completeness of the article, our experiment includes a group that knocked down AlkBH2, whose effect may be like its inhibitor. This should be able to contrast sharply with the effects observed from AlkBH2 overexpression, highlighting the role of AlkBH2 in bladder cancer.

7. Thank you for your suggestions regarding the quality of some of the images. In order to improve the image quality, we conducted new cloning experiments and Transwell experiments. However, for the scratch experiment, due to the limitations of the microscope, it was difficult to capture a very clear image using white light. Therefore, we used black-and-white images to highlight the shape of the scratch.

8. Thank you for your feedback. To ensure the reproducibility of the experiment, we have provided the information of the key reagents, including antibodies, cell lines, and the manufacturer names, product numbers of the key reagents, in the attachment.

9. Thank you very much for your suggestions. We have revised some of the materials and methods, especially regarding the analysis plan for the cell cycle and the detailed steps of the angiogenesis experiment, such as cell density, pre-treatment duration, serum conditions, and standardization strategies, etc., all of which have been clearly clarified.

10. Thank you very much for your suggestion. We have reorganized the materials and methods section to match the sequence of the "results" section.

11. Thank you very much for your suggestion. We have revised the discussion section and streamlined it. We focused on targeted discussions, directly connecting the key findings of the research with existing literature, while clearly highlighting its novelty and limitations.

12. Thank you very much for your feedback. We sought assistance from native English speakers and made revisions to the English grammar to make it more in line with the requirements for publication in the magazine.

13. Thank you very much for your suggestion. We have defined the first occurrence of the abbreviation.

14. Thank you very much for your feedback. We have carefully standardized the formats of superscripts, subscripts and symbols.

To reviewer2

Thank you for your suggestions, which is very helpful in improving the quality of this article.

1. Thank you very much for your suggestion regarding observing the effect of this gene on inflammation after mutation in AlkBH2. We totally agree with this method directly proved the relationship between AlkBH2 and validation. However, due to the limitations of funds and time, we only verified the above relationship from a side perspective. In Figure 5, overexpression of AlkBH2 clarified that AlkBH2 promotes inflammation in bladder cancer; while knocking down the AlkBH2 gene inhibited the inflammatory response, once again demonstrating the relationship between AlkBH2 and tumor inflammation. We hope that the "knockdown experiment" can, to certain extent, replace the AlkBH2 mutation experiment and make up for the shortcomings of this study. Of course, we also indicated our shortcomings in the article.

2. Thank you very much for your suggestion regarding the proteomics results. Due to the unsatisfactory outcome of the proteomics sequencing, we did not include it. Now, after listening to your suggestions, we have placed the volcano plot, heat map, and enrichment plot, among others, into the figures. This provides strong evidence to prove the validity of the statement that AlkBH2 activates the NF-κB pathway.

3. Thank you very much for your feedback. We carefully standardized the formatting for different sizes of fonts.

4. Thank you very much for your feedback. DAPI is present in the picture, but it needs to be enlarged for a clear view. Readers can enlarge the pictures for reading without being affected by this. The reason why it was not immediately found at first glance is as follows: 1) The third group is the AlkBH2 knockdown group. Under the same time conditions, cell growth was significantly inhibited, so it was very scarce. 2) To compare at the same level, the fluorescence shooting conditions cannot be changed, so the knockdown group with fewer cells had to be sacrificed, resulting in a relatively reduced absorption of blue light. In the future, we will overcome these limitations and improve our research techniques.

5. Thank you very much for your feedback. To ensure the reproducibility of the experiment, the information of key reagents has been provided, including antibodies, cell lines, and the manufacturer names, product numbers of the key reagents, as well as the relevant primer sequences used for PCR, which are included in the attachment.

6. Thank you very much for your comments. The silent and overexpressed lentiviruses were purchased from the company. We have indicated in the methods that they were obtained from GenePharma Company.

7. Thank you very much for your suggestion. We have incorporated the content you provided into the preface. To make the text more readable for the readers, we have revised the introduction and discussion sections, simplifying them and directly connecting the key findings of the research with the existing literature. At the same time, we have clearly highlighted its novelty and limitations.

At last, based on the opinions of the editors and reviewers, we carefully answered each question one by one, and made every effort to supplement additional experiments and provide explanations to address the relevant issues in order to improve the quality of the article. We hope to meet the requirements of the journal and be successfully accepted.

Best wishes,

Prof Chen
