# Supplementary material for: A new target: AlkBH2 promotes bladder cancer by upregulation of inflammation
Source: PLoS One. 2026 May 7;21(5):e0348059. doi: 10.1371/journal.pone.0348059 (PMC13152141; doi:10.1371/journal.pone.0348059)
Supplement: Attachment — Submitted filename: Response_to_Reviewers_auresp_2.docx [file pone.0348059.s005.docx]

Dear editor,

Thank you very much for your recognition of our work. We have made detailed revisions according to your suggestions to meet the requirements of your journal.

1. According to your advice, we have uploaded original data to Protocols.io. (DOI)：https://www.protocols.io/file/be2wqczccx.zip

2. We are verifying our ORCID iD within the submission system before final publish.

To reviewer1

Thank you for all the comments you have made on our article. Your opinions not only corrected our mistakes but also significantly improved the quality of the article.

1. I'm very sorry for my oversight that caused you to misunderstand. In the last response, we clearly stated that we had added mitomycin C. However, because English is not our native language, we used "without" in the article revision, while our original intention was "with". Well, I admit that I am a rather careless person. Once again, thank you for your carefulness and rigor in pointing out our mistakes.

2. Thank you for your feedback. Standardized CM refers to the preparation of a standard mixed culture medium containing factors secreted by bladder cancer cells, which is used to stimulate HUVEC and evaluate its angiogenic promoting ability. Here, "standardized" means that we use a unified cell count of 10^6^ and uniformly culture for 48 hours to obtain the mixed culture medium containing the factors secreted by bladder cancer cells. The corresponding explanation has been supplemented in the methods section.

3. Thank you very much for your question. All our groups, including the control group and the ALKBH2+ and ALKBH2- groups, were transfected with lentivirus carrying the green fluorescent protein gene (GFP). The difference is that the control group was only transfected with the GFP gene, while the ALKBH2+ and ALKBH2- groups were transfected with the GFP gene in addition to overexpressed and interfered genes. Therefore, all three groups of cells have green fluorescence that were observed in Fig 2A, in order to ensure the consistency of the experiment. The corresponding explanations have been supplemented in the methods section.

4. Thank you very much for your suggestion. In the real-time cell proliferation experiment, a mean ± SD value is obtained at extremely short time intervals. In Fig 3A, we plotted the graph based on each mean ± SD value. By zooming in, we can observe that it is composed of countless independent mean ± SD points and lines. The original data including mean ± SD value has been uploaded to the editing department. After the article is officially published, readers should be able to access it through the link.

5. Thank you very much for your suggestion. The reason why the bar graphs in Fig 5 are not uniform is due to the layout issue. If all were vertical bars, it would not be possible to place the ELISA results in one figure. Considering the logic and continuity of the article, we did our best to place the ELISA results in one figure, which led to some bar charts having to be horizontal bars. At the same time, we ingeniously distinguished the types of inflammatory factors through different types. All vertical bars represent pro-inflammatory factors, while the horizontal bars represent factors that inhibit inflammation. This not only solves the layout issue but also enhances the readers' interest in reading to a certain extent.

6. Thank you very much for your feedback. We originally thought that listing only the main reagents would suffice. We have now provided detailed information about all the reagents in Table S1.

To reviewer2

Thank you very much for your appreciation of this article. Your recognition and encouragement are the greatest motivation for our progress. Thank you again!

Best wishes,

Prof Guojun Chen
